# Screening value of the Center for epidemiologic studies – depression scale among people living with HIV/AIDS in Ho Chi Minh City, Vietnam: a validation study

**DOI:** 10.1186/s12888-016-0860-3

**Published:** 2016-05-13

**Authors:** Truc Thanh Thai, Mairwen K. Jones, Lynne M. Harris, Robert C. Heard

**Affiliations:** Faculty of Public Health, Ho Chi Minh City University of Medicine and Pharmacy, 159 Hung Phu Street, District 8, Ho Chi Minh City, Vietnam; Faculty of Health Sciences, University of Sydney, 75 East Street, Lidcombe, Sydney, 2141 Australia; School of Psychological Sciences, Australian College of Applied Psychology, Level 6, 11 York Street, Sydney, 2000 Australia

**Keywords:** CES-D, Depression, HIV outpatient, Validation

## Abstract

**Background:**

Depression is believed to be under-diagnosed and under-treated in people living with HIV/AIDS (PLHIV). Early screening and referral to mental health services for treatment has been shown to enhance HIV patients’ health during the course of HIV treatment. A lack of psychiatric specialist services for PLHIV at outpatient clinics (OPC) in Vietnam leads to insufficient identification of depression. However, alternative approaches are available such as the use of screening scales. This study investigated the psychometric properties of the Center for Epidemiologic Studies – Depression scale (CES-D) in Vietnamese HIV positive outpatients.

**Methods:**

A cross-sectional survey of 400 HIV positive outpatients was conducted in Ho Chi Minh City, Vietnam. Participants completed a self-reported questionnaire that included the CES-D. Participants were also interviewed independently by a psychiatrist who assessed for symptoms of major depressive disorder. CES-D reliability was measured by Cronbach’s alpha. Criterion validity was evaluated by ROC analysis, Kappa index and the percentage of agreement between the CES-D and psychiatrists’ interview. Construct validity was investigated by confirmatory factor analysis.

**Results:**

The reliability for the whole scale was good (Cronbach α = 0.81). The four sub-scales of the CES-D had lower levels of internal consistency with Cronbach alpha of 0.71, 0.73, 0.71 and 0.58 for somatic complaints, depressive affect, positive affect and interpersonal problems respectively. CES-D has adequate construct validity with CFI = 0.926, IFI = 0.927, GFI = 0.930 and RMSEA = 0.045 (90 % CI = 0.037–0.053) in the final four-factor model. Area under curve was 0.88 indicating good criterion validity. At the cutoff of 16, the sensitivity and specificity were 79.8 % and 83.0 % respectively while the percentage of agreement between the CES-D and psychiatrists’ interview was 82.0 % with Kappa index at 0.60.

**Conclusions:**

The CES-D was shown to be acceptable, reliable and valid for screening symptoms of depression in Vietnamese HIV outpatient clinic settings where mental health specialists are not always available. Routine use of the CES-D at HIV outpatient clinics, in combination with the availability of free-for-all national mental health services, is likely to be beneficial in improving the lives of PLHIV in Vietnam who have depression.

**Electronic supplementary material:**

The online version of this article (doi:10.1186/s12888-016-0860-3) contains supplementary material, which is available to authorized users.

## Background

The first person identified as HIV positive in Vietnam was reported in 1990. In 2013, it was estimated that nearly 248,500 people are living with HIV/AIDS in Vietnam [1]. With support from national and international programs, the HIV epidemic in Vietnam is believed to be under control as the incidence of HIV has been stable since 2012 [1, 2]. The success of anti-retroviral treatment programs has resulted in people living with HIV/AIDS (PLHIV) living longer and attention has turned to addressing the related health problems that commonly affect PLHIV [3]. The most common non-infectious conditions experienced by PLHIV are mental health problems, particularly depression [4]. Despite the high prevalence of depression among PLHIV reported worldwide, estimated at about 40–42 % [4], mental health problems such as depression are believed to be under-diagnosed and under-treated in PLHIV in developing countries like Vietnam [5]. Studies conducted in Vietnam have reported depression prevalence between 18.7 % among men living with HIV using the Phan Vietnamese Psychiatric Scale [6], 40 % in outpatients using a scale designed for the study [3], and up to 47.3 % among men who have sex with men using the short version of the Center for Epidemiologic Studies – Depression scale (CES-D) [7].

One possible explanation for this is the lack of psychiatric specialist services for PLHIV at outpatient clinics (OPC) in Vietnam [8] which leads to insufficient identification of depression by clinic health staff who are not trained to recognise symptoms of depression. It was estimated that there were about 286 psychiatrists in Vietnam in 2004 and that none of these psychiatrists worked at outpatient facilities [8]. While financial considerations may limit the availability of specialist psychiatrists or training of OPC staff to identify symptoms of mental illness such as depression, alternative less costly approaches are available such as mental health screening scales which have been developed and are widely used [9]. These provide a cost-effective means by which non-specialist staff can identify people requiring referral to psychiatric services and are therefore very appropriate for use in resource-limited countries such as Vietnam [9, 10]. Early screening and referral to mental health services for treatment has been shown to enhance PLHIV’s health during the course of HIV treatment [10, 11]. The standard inclusion of a screening scale for depression at HIV OPCs would guide referral to existing, free, national mental health services, thus improving the lives of PLHIV in Vietnam who have depression.

One of the most frequently used screening tools for depression is the Center for Epidemiologic Studies – Depression scale (CES-D), a 20 item self-report instrument designed to screen for depression in primary care settings [12]. The CES-D has also been used extensively to assess depression in people with chronic diseases [13], including HIV positive patients [14] especially in high HIV burden settings [15, 16]. The CES-D has high internal consistency, with Cronbach’s α = 0.85 for the general population and 0.90 for patient samples, and adequate test-retest repeatability [12]. The criterion validity of the CES-D has been assessed with clinician’s rating of depression, and on relationships with other factors that support its construct validity [12].

It is acknowledged that self-report screening scales such as the CES-D may be affected by the context in which they are used, and that translation of the original English language version into Vietnamese may also change the meaning of the test [17, 18]. Differences in ethnicity, culture and language are likely to influence the way people understand and describe symptoms of mental illness [19, 20] and may affect willingness to disclose symptoms of mental illness, especially in Asian communities [19, 21]. No scale can be expected to be suitable for all populations or completely culture-free so that the psychometric properties of the CES-D may be different in specific target populations to which the scale is applied [17]. Even among people of the same ethnicity it is likely that sub-groups, such as people with diverse health conditions, might respond differently to a questionnaire. As such, the psychometric properties of the scale may differ from those found in the broader population [17, 22]. Variation in the psychometric properties of the same version of the CES-D has been reported among samples of undergraduates, community dwelling adults, people receiving rehabilitation for injuries and women with major depressive disorder [22]. Therefore, evaluation of the psychometric properties of the translated CES-D is necessary before introducing it as a standard screening tool for people from different cultures, language backgrounds and/or with specific health conditions.

A Vietnamese translation of the CES-D has been developed in the United States among Vietnamese communities [23, 24] and has been used with community-based Vietnamese adults and in school-based adolescents in Vietnam [25, 26]. However, the performance of this instrument in Vietnam among PLHIV has not been evaluated. In light of the need for a brief screening instrument for symptoms of depression for use with PLHIV in Vietnam, the present study investigated the psychometric properties of the CES-D, including internal consistency, criterion-related validity and construct validity among PLHIV in Vietnam. The findings of this study will inform decision-making concerning the use of the CES-D as a screen for symptoms of depression among PLHIV in Vietnamese OPCs.

## Methods

### Setting and participants

Ho Chi Minh City has a population of approximately eight million people [27] and is also a ‘hot spot’ for HIV/AIDS in Vietnam with almost 25,000 PLHIV receiving care and treatment at 30 OPCs in 2011 [1]. The study employed a cross-sectional survey methodology. An average of 1,000 PLHIV attend each of the two randomly selected OPCs every month and one in five PLHIV were consecutively invited to participate in the study when attending their regular monthly appointment. The sample size was originally calculated to estimate prevalence of mental disorders. With estimated prevalence of 50 %, type 1 error rate of 5 % and the width of the 95 % confidence interval of 10 %, at least 385 PLHIV were needed. Over a four week period during November and December 2013, 410 who were eligible were approached. Eligibility criteria were ≥ 18 years old, currently receiving care at the study OPC and able to read and write. Of these 400 PLHIV (200 from each OPC) participated and completed the survey.

### Procedure and measurement

Selected participants were provided with an information statement and a consent form. After reading the information statement those who did not want to participate continued with their usual clinic care. Those who chose to participate signed the consent form and were then directed to a private room and asked to complete a self-report questionnaire which included the CES-D. A research assistant was in the room to answer any questions raised. Participants sealed their completed questionnaires in an envelope and placed the envelope in a box so as to ensure confidentiality. Participants were then assessed by a psychiatrist, who did not have access to questionnaire responses, to determine whether they had symptoms of depression based on the Diagnostic and Statistical Manual of Mental Disorders, 4th Edition, Text Revision (DSM-IV-TR) [28] criteria for major depressive disorder. Participants with symptoms of depression identified by the psychiatrist as requiring specialist referral and treatment were classified as positive for depression and referred to mental health services for free treatment. Clinical information was also extracted from medical records upon participants’ approval in the consent form, where participants provided their patient identification number. Data on the questionnaire and results from the psychiatrists’ interview were matched using a pre-defined study identification number printed on the forms and questionnaire so that no identifying information about the patient was stored.

#### Background information

The questionnaire included items about gender, sexual orientation, age, highest level of education, employment status, economic status, religion, marital status, parental status, and source of HIV infection. Time since HIV diagnosis and number of family members who had been diagnosed HIV positive were extracted from medical records.

#### Center for epidemiologic studies - depression scale (CES-D)

The 20-item CES-D measures depression symptoms during the past week. Participants rate the CES-D items using a Likert rating system ranging from 0 (rarely or none of the time/less than 1 day) to 3 (most or all of the time/5–7 days). The total score ranges from 0 (no symptoms of depression) to 60 (have almost all symptoms of depression). Factor analysis conducted with samples of general population and psychiatric patients indicates that the CES-D has four factors: depressed affect, positive affect, somatic complaints and interpersonal problems [12]. Although the CES-D was available in the Vietnamese language in previous studies [23, 24, 29], the original version was also translated into Vietnamese independently both by the first author and an accredited translator. Differences between researcher and the accredited translator were discussed. The revised version was compared to the previously translated versions. No changes were made to the content of the items during the final comparison except that “during the last week” was added for each item to ensure that respondents focused on this time frame so as to avoid recall bias (Additional file 1).

### Data imputation

For missing data from the CES-D scale, the mean of the item for all participants’ response was used. Although this method may result in lack of variance in the sample and the observed correlation, there is no evidence that this results in differences in terms of estimation compared to other methods including person mean imputation, regression imputation and hot-deck imputation [30]. Given the small amount of missing data in the present study, this item-mean imputation was therefore expected to convey no disadvantage [30]. Eighteen returned questionnaires (4.5 %) had missing values for the CES-D, including 15 questionnaires with one item not completed and three questionnaires with two items not completed. These items were imputed by taking the mean of that item for all participants before conducting further analysis. The missing items were evenly spread across the questionnaire.

### Data analysis

Counts and percentages were used for descriptive analysis for categorical variables while differences in proportion of symptoms of depression among participants were evaluated using the Chi-squared statistical test or the Fisher’s exact test as appropriate. Means and standard deviations were employed as descriptive statistics for quantitative variables and the differences in means between those with and without depression symptoms were analyzed using independent *t* test. Scores for CES-D and subscales adjusting for gender, parental status, economic status and time since HIV diagnosis were determined by fitting a linear regression model. The type 1 error rate was set at 0.05.

The reliability of the CES-D was measured with the Cronbach alpha and item-test, item-rest correlation coefficients. The Cronbach alpha measures the extent to which the items consistently measure the same thing [31]. The item-test correlation indicates whether the response of every item is consistent with averaged behavior of the test while the item-rest reveals the strength of correlation between item score and the scores of the other items as a whole. These correlation coefficients are similar to Pearson’s correlation coefficient which ranges from 0 to 1; the higher the value they have, the more consistent the item is [17]. Although a Cronbach alpha coefficient of 0.80 is considered as a reasonable benchmark indicating good internal consistency, in many cases, if this statistic is equal or greater than 0.70 then internal consistency is considered adequate [31, 32]. An alpha of 0.60 is acceptable in some cases [33].

Construct validity was examined using confirmatory factor analysis (CFA) based on the originally established four-factor model [12]. The Chi-squared statistic was used to identify whether the model fit the data well. However, as the Chi-squared statistic is influenced by sample size, where model fit is poorer with larger sample size [34], the Comparative Fit Indices (CFI), Goodness of Fit Indices (GFI), Incremental Fit Indices (IFI) and Root Mean Square Error of Approximation (RMSEA) were also assessed. The CFI and IFI compare the model with alternative models such as a null model or independence model where variables are assumed to have zero correlation, while the GFI reveals the discrepancy between the hypothesized model and the observed covariance matrix [35, 36]. The values of CFI, GFI and IFI > 0.90 indicate a well-fitting model [35, 36]. The Root Mean Square Error of Approximation (RMSEA) with a 90 % confidence interval indicates “badness of fit” and thus the smaller the RMSEA, the closer the fit between model and data [37]. A RMSEA value <0.05 indicates a good fit [38] and its 90 % confidence interval is expected not to exceed 0.08 [36].

For criterion validity, the decision of the psychiatrists was treated as the gold standard and thus ROC analysis was conducted to evaluate sensitivity, specificity and area under the curve for common cut-off points suggested in previous studies (i.e. 16, 21) [13, 14, 20, 39]. For a screening test, sensitivity is expected to be at least 80 % to minimize the false negative cases while the area under the curve should be at least 0.80 to have correct classification of PLHIV with and without depression symptoms using the scale. Kappa statistics and percentage of agreement between CES-D score and the psychiatrists’ decision were also calculated. Kappa indicates whether there is agreement between CES-D and psychiatrists’ interview result and can be categorized as poor (<0.20), fair (0.20–0.40), moderate (0.41–0.60), good (0.61–0.80) and very good (0.81–1.00) [40, 41]. All analyses were conducted using AMOS and SPSS version 21 [42].

### Ethics

All procedures in this study were approved by the Ho Chi Minh City Provincial AIDS Committee Human Ethics Committee (Number IRB-03-2013, dated 17/10/2013) and the University of Sydney Human Research Ethics Committee (Number 2013/859, dated 15/11/2013).

## Results

### Participant characteristics and major depressive disorder

Most participants were male (63.5 %) and the mean age was 34.8 years (SD = 6.8 years). The main reported reason for HIV infection was sexual transmission (56 %) and the mean time since HIV diagnosis was 5.2 years (SD = 2.5 years). Thirty one percent of PLHIV were identified by psychiatrists as having symptoms of depression. There was no significant association between presence of depression symptoms and sexual orientation, age, work status, education level, marital status, parental status, religion, economic status, source of HIV infection and having family member been diagnosed HIV positive (see Table 1). However, there was a significant association between presence of depression symptoms and gender, where the percentage of females with symptoms of depression was higher (40.4 %) than the percentage of males with symptoms of depression (25.6 %) and the average time from HIV diagnosis was lower in those who had symptoms of depression than those who did not.

**Table 1:** Characteristics of people living with HIV/AIDS, stratified by depression symptoms identified by psychiatrists’ interview (*N* = 400)

Characteristics	Depression symptoms		*p*-value^a^
	Yes (*N* = 124, 31 %) *n* (%)	No (*N* = 276, 69 %) *n* (%)	
Gender			
Male	65 (52.4)	189 (68.5)	0.002
Female	59 (47.6)	87 (31.5)	
Sexual orientation			
Heterosexual	108 (87.0)	243 (88.1)	0.922
Homosexual/Bisexual	8 (6.5)	15 (5.4)	
Unsure/Not answer	8 (6.5)	18 (6.5)	
Age, yrs [M (SD)]	34.8 (7.6)	34.9 (6.5)	0.927^b^
Highest level of education			
Did not complete primary	19 (15.3)	34 (12.3)	0.710
Primary school	22 (17.7)	64 (23.2)	
Secondary school	46 (37.1)	105 (38.0)	
High school	30 (24.2)	59 (21.4)	
College or more	7 (5.7)	14 (5.1)	
Work status			
Unemployed	31 (25.0)	53 (19.2)	0.354
Casual job	24 (19.4)	44 (16.0)	
Part-time job	21 (16.9)	48 (17.4)	
Full-time job	31 (25.0)	95 (34.4)	
Other	17 (13.7)	36 (13.0)	
Self-rated economic status			
Very poor/Poor	62 (50.0)	112 (40.6)	0.061^c^
Average	60 (48.4)	163 (59.1)	
Rich/Very rich	2 (1.6)	1 (0.3)	
Have a religion			
Yes	31 (25.0)	73 (26.4)	0.760
No	93 (75.0)	203 (73.6)	
Marital status			
Single	32 (25.8)	92 (33.3)	0.262
Married/Live as a couple	75 (60.5)	144 (52.2)	
Divorced/Separated/Widowed	17 (13.7)	40 (14.5)	
Parental status			
Yes	78 (62.9)	141 (51.1)	0.028
No	46 (37.1)	135 (48.9)	
Source of HIV infection			
Sexual transmission	72 (58.1)	152 (55.1)	0.467
Injected drug use	32 (25.8)	87 (31.5)	
Others	20 (16.1)	37 (13.4)	
Time since HIV diagnosis, yrs [M (SD)]	4.8 (2.6)	5.4 (2.5)	0.033^b^
Family members been diagnosed HIV positive			
None	79 (63.7)	199 (72.1)	0.123^c^
One	43 (34.7)	69 (25.0)	
Two or more	2 (1.6)	8 (2.9)	

^a^Chi-squared test unless otherwise stated

^b^t test

^c^Fisher exact test

### Reliability

Table 2 shows the percentage of respondents endorsing each response option for each CES-D item. The mean, correlation coefficients and Cronbach’s alphas were calculated after missing data was imputed. The CES-D has moderate to strong item-test correlation with coefficients ranging from 0.28 to 0.65 and reasonable to good item-rest correlations, with coefficients between 0.14 and 0.58. The internal consistency for the whole scale was good (Cronbach α = 0.81), and the values of Cronbach alpha in the absence of each individual item ranged from 0.80 to 0.83 indicating that all items should be retained. The four sub-scales of the CES-D had lower levels of internal consistency with Cronbach alpha of 0.71, 0.73, 0.71 and 0.58 for somatic complaints, depressive affect, positive affect and interpersonal problems respectively (see Table 3).

**Table 2:** Item distribution and internal consistency of the CES-D in people living with HIV/AIDS (*N* = 400)

Item	Score [*n* (%)]^a^				M (SD)	Item-test correlation	Item-rest correlation	Alpha if item deleted
	0	1	2	3				
I was bothered by things that don’t usually bother me	229 (57.3)	101 (25.3)	56 (14.0)	14 (3.5)	0.6 (0.9)	0.59	0.51	0.80
I did not feel like eating; my appetite was poor	190 (47.7)	119 (29.9)	71 (17.8)	18 (4.5)	0.8 (0.9)	0.55	0.47	0.80
I felt that I could not shake off the blues even with the help of my family or friends	283 (71.1)	76 (19.1)	30 (7.5)	9 (2.3)	0.4 (0.7)	0.55	0.48	0.80
I felt that I was just as good as other people^b^	91 (22.8)	65 (16.2)	127 (31.8)	117 (29.2)	1.3 (1.1)	0.28	0.14	0.83
I had trouble keeping my mind on what I was doing	207 (51.9)	116 (29.1)	60 (15.0)	16 (4.0)	0.7 (0.9)	0.43	0.34	0.81
I felt depressed	235 (58.9)	92 (23.1)	60 (15.0)	12 (3.0)	0.6 (0.8)	0.60	0.52	0.80
I felt everything I did was an effort	230 (57.5)	94 (23.5)	40 (10.0)	36 (9.0)	0.7 (1.0)	0.40	0.30	0.81
I felt hopeful about the future^b^	118 (29.6)	97 (24.3)	110 (27.6)	74 (18.5)	1.6 (1.1)	0.31	0.18	0.82
I thought my life had been a failure	255 (63.8)	86 (21.5)	42 (10.5)	17 (4.2)	0.6 (0.8)	0.46	0.37	0.81
I felt fearful	300 (75.8)	64 (16.1)	30 (7.6)	2 (0.5)	0.3 (0.6)	0.44	0.37	0.81
My sleep was restless	213 (53.4)	103 (25.8)	63 (15.8)	20 (5.0)	0.7 (0.9)	0.57	0.48	0.80
I was happy^b^	46 (11.6)	88 (22.1)	144 (36.2)	120 (30.1)	1.2 (1.0)	0.57	0.48	0.80
I talked less than usual	246 (61.5)	86 (21.5)	51 (12.8)	17 (4.2)	0.6 (0.9)	0.54	0.46	0.80
I felt lonely	253 (63.3)	83 (20.7)	53 (13.3)	11 (2.7)	0.6 (0.8)	0.60	0.53	0.80
People were unfriendly	305 (76.8)	60 (15.1)	25 (6.3)	7 (1.8)	0.3 (0.7)	0.49	0.43	0.81
I enjoyed life^b^	47 (11.8)	57 (14.2)	132 (33.0)	164 (41.0)	1.0 (1.0)	0.36	0.25	0.82
I had crying spells	326 (81.7)	44 (11.0)	20 (5.0)	9 (2.3)	0.3 (0.7)	0.42	0.35	0.81
I felt sad	208 (52.4)	116 (29.2)	55 (13.9)	18 (4.5)	0.7 (0.9)	0.65	0.58	0.80
I felt that people disliked me	347 (86.8)	44 (11.0)	9 (2.3)	0	0.2 (0.4)	0.43	0.39	0.81
I could not get “going”	297 (74.3)	66 (16.5)	30 (7.5)	7 (1.8)	0.4 (0.7)	0.44	0.36	0.81

^a^0 = Rarely or none of the time (<1 day); 1 = Some or a little of the time (1–2 days); 2 = Occasionally or a moderate amount of the time (3–4 days); 3 = Most or all of the time (5–7 days)

^b^Items were reversed before calculating mean, median, correlation and alpha

Item-means were used to calculate score for item (number of missing): 2(2), 3(2), 5(1), 6(1), 8(1), 10(4), 11(1), 12(2), 15(3), 17(1), 18(3)

**Table 3:** Internal consistency of the CES-D and subscales in people living with HIV/AIDS (*N* = 400)

Scales	Items	Alpha	M (SD)	Depression symptoms^a^		*p*-value
				Yes (*N* = 124) M (SD)	No (*N* = 276) M (SD)	
CES-D	20	0.82	13.6 (8.0)	21.0 (7.0)	10.2 (6.0)	<0.001
Somatic complaints	7	0.71	4.5 (3.6)	7.7 (3.4)	3.1 (2.7)	<0.001
Depressive affect	7	0.73	3.5 (3.4)	6.3 (3.2)	2.2 (2.6)	<0.001
Positive affect	4	0.71	5.1 (3.1)	6.0 (2.7)	4.7 (3.2)	<0.001
Interpersonal problems	2	0.58	0.5 (0.9)	1.0 (1.2)	0.3 (0.7)	<0.001
Adjusted CES-D^b^				21.0 (1.0)	10.2 (1.0)	<0.001
Somatic complaints^b^				7.8 (0.5)	3.1 (0.5)	<0.001
Depressive affect^b^				6.3 (0.5)	2.2 (0.5)	<0.001
Positive affect^b^				6.0 (0.4)	4.7 (0.3)	<0.001
Interpersonal problems^b^				1.0 (0.1)	0.3 (0.1)	<0.001

^a^Identified by psychiatrists’ interview

^b^Adjusted for gender, economic status, parental status and time since HIV diagnosis

### Construct validity

**Fig. 1:**
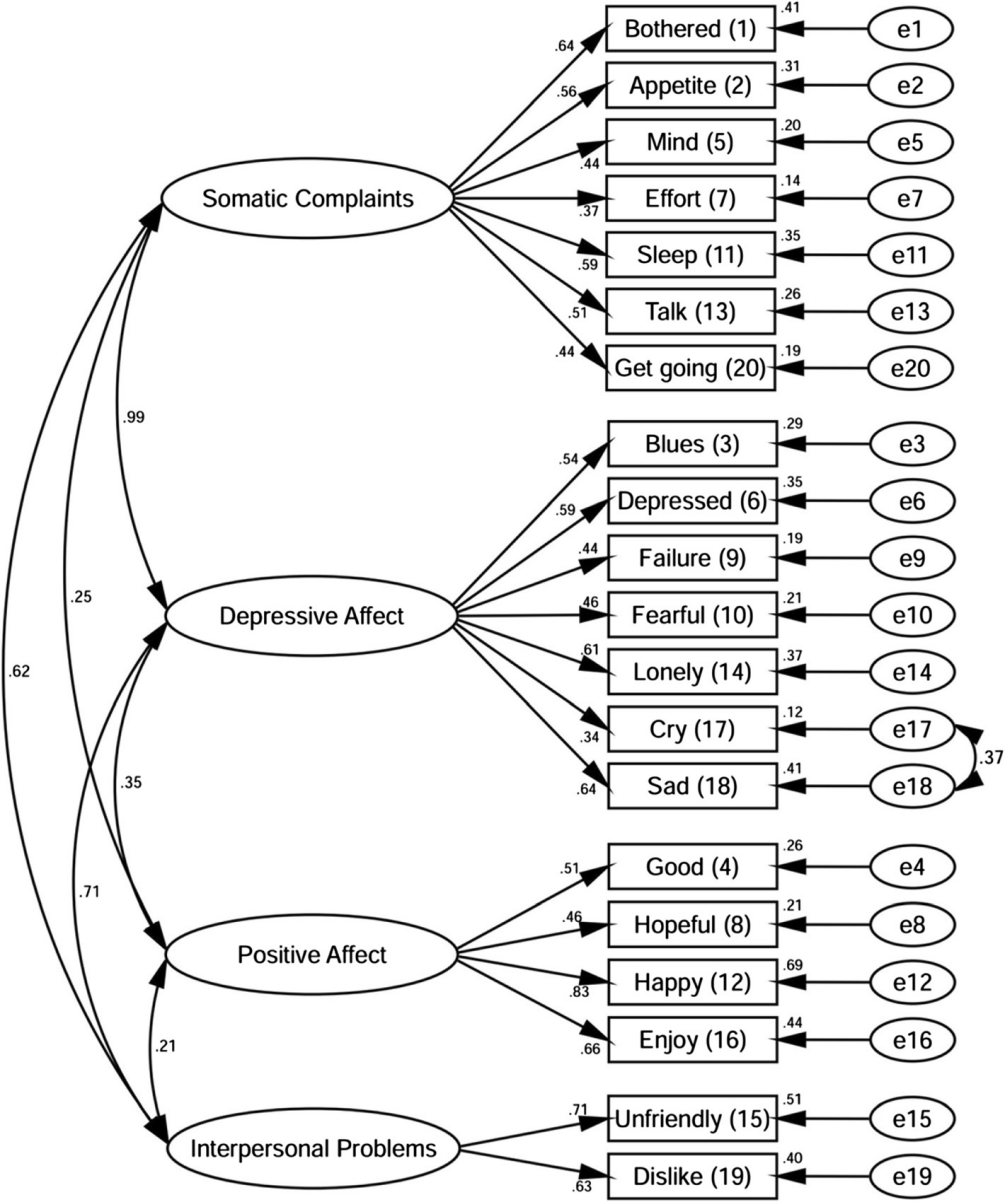
Confirmatory factor analysis for CES-D in people living with HIV/AIDS (*N* = 400)

The model fit indices in the original four-factor model indicated a marginal fit with CFI = 0.898, IFI = 0.900, GFI = 0.919 and RMSEA = 0.052 (90 % CI 0.045–0.060). The modification indices suggested specifying a covariance between the errors of item 17 (crying) and 18 (sad) would improve the model fit. Therefore, in the final model a covariance between the errors of these two items was specified (see Fig. 1). The confirmatory factor analysis showed a significant difference between the observed model and expected model using the Chi-squared statistics fit index (*χ*^2^ = 294.12, df = 163, *p* < 0.001). However, as noted above, the Chi-squared is likely to be affected by the large sample size. The CFI (0.926), IFI (0.927), GFI (0.930), and RMSEA (0.045; 90 % CI = 0.037–0.053) all revealed a good fit for the model specified. The four factors had moderate to very strong correlations with one another, with correlation coefficients ranging from 0.21 to 0.99. Factor loadings ranged from 0.34 to 0.83. Multiple squared correlation coefficients ranged from 0.12 to 0.69 indicating the factors provide an acceptable to good explanation of variation found in the items used in the CFA model. Together, these data indicate that the CES-D has adequate construct validity.

### Criterion validity

**Fig. 2:**
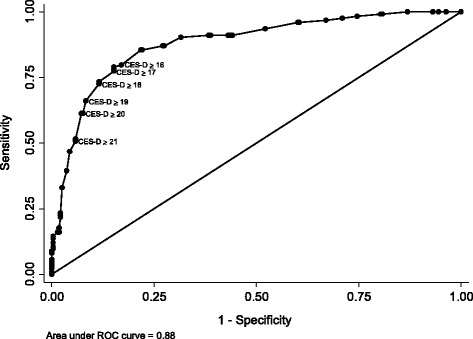
Characteristics performance of the CES-D in people living with HIV/AIDS (*N* = 400)

The scores of CES-D and all subscales were significantly different among those with and without depression symptoms from psychiatrists’ interview (Table 3). The differences statistically persisted after adjusting for gender, parental status, economic status and time since HIV diagnosis. The shape of the ROC curve and the area under the curve (AUC = 0.88) indicated good criterion validity of the CES-D using the total score (see Fig. 2). As such, the CES-D can be used to screen PLHIV with symptoms of depression from those without. Previous international studies have established cutoff scores ranging between 16 and 21. All scores in this range were tested as potential cutoffs (see Table 4). Overall, an increase of the cut-off point is likely to result in decreased sensitivity and increased specificity. Table 4 suggests 16 is an optimal cutoff at which the sensitivity and specificity were 79.8 and 83.0 respectively, while the AUC was good at 0.81. With a score of 21, the CES-D had lower sensitivity (50.8) but a much higher specificity (94.2). Despite the change of sensitivity and specificity using different cut-off points, the percentage of agreement with the psychiatrists’ interview was quite stable ranging from 80.1 % to 83.8 %, and the Kappa index ranging from 0.50 to 0.61 indicated good agreement between the CES-D and the psychiatrists’ decision.

**Table 4:** CES-D performance properties for different cut-off points in people living with HIV/AIDS (*N* = 400)

Cutoff	Prevalence	Sensitivity	Specificity	AUC	% Agreement^a^	Kappa
≥16	36.5	79.8	83.0	0.81	82.0	0.60
≥17	34.5	77.4	84.8	0.81	82.5	0.60
≥18	30.5	72.6	88.4	0.80	83.5	0.61
≥19	26.3	66.1	91.7	0.79	83.8	0.60
≥20	24.0	61.3	92.8	0.77	83.0	0.58
≥21	19.8	50.8	94.2	0.73	80.1	0.50

^a^Agreement with psychiatrists’ interview

## Discussion

Overall, the study shows high prevalence of symptoms of depression assessed both by psychiatrists’ interview and by the self-report CES-D scale. In this study, the CES-D had high levels of reliability by Cronbach alpha, construct validity by confirmatory factor analysis and criterion validity by ROC analysis. The CES-D was also revealed to be well accepted with a low level of missing data.

Based on the psychiatrists’ interview approximately one third (31 %) of the Vietnamese PLHIV who participated in this study experienced symptoms of depression. This is more than 10 times higher than the prevalence of depression in the general population in Vietnam. The prevalence of depression from a national community-based survey conducted by National Psychiatric Hospital Number 1 was about 2.8 %, although the assessment method was not reported [8]. Despite the different methods used, the prevalence of depression found in this study and the prevalence of 40 % reported by Green et al. [3] confirm that many Vietnamese PLHIV are also living with symptoms of depression that warrant referral to specialist mental health services. Vietnam is a resource-limited country where specialist mental health services are not routinely available at OPCs. Thus, the availability of a validated screening scale, that can be used by staff without mental health training to identify those who require referral to services under the Vietnam national mental health program, has the potential to improve the quality of life for a significant minority of Vietnamese PLHIV, since early detection of depression can lead to appropriate, effective intervention [10, 11].

In the current study, the CES-D had very good reliability similar to that reported for the original version [12] and reasonably similar to studies employing this scale in adolescents and young adults without HIV in Vietnam, where Cronbach’s alpha has been reported to range from 0.78 to 0.86 [25, 26, 29, 43]. Compared to other validation studies, especially in non-US, high HIV burden settings, the Cronbach’s alpha in this study was similar to a self-report-based study in tuberculosis and HIV patients in Zambia (α = 0.84) [15] but lower than an interview-based study among pregnant women with and without HIV in Uganda (α = 0.92) [16]. However, the two-item ‘interpersonal problems’ subscale had very low internal consistency, as often occurs with scales that have fewer items [34]. The low Cronbach’s alpha found in this study was consistent with that found in a previous study among medically ill elderly by Schein and Koenig [44], where in the study by Schein and Koenig [44] the ‘interpersonal problems’ subscale had the lowest Cronbach alpha coefficient (α = 0.40).

In terms of construct validity, although the Chi-squared test indicated that the model was not a good fit in this sample, the four other fit indices used here indicated that the CES-D data was a good fit to a 4-factor model (somatic complaints, depressive affect, positive affect and interpersonal problems). The poor fit indicated by the Chi-squared statistic is likely to be due to the large sample size used here [36]. In sample sizes larger than 200, the Chi-squared test is known to be inflated and to yield significant findings [36, 45]. The findings of the present study are consistent with those of previous studies where the four-factor model did not satisfy the Chi-squared test but the CFI, IFI and GFI all showed good fit [46]. Overall, the CES-D can be considered to have adequate construct validity. Furthermore, the correlation between the four factors in the model was similar to that reported by McCallum et at [46], where in the study by McCallum et al. [46] somatic complaints and depressive affect had the highest correlation coefficients (r = 0.90) followed by depressive affect and interpersonal problems (r = 0.69) and somatic complaints and interpersonal problems (r = 0.61). These figures also indicate that the “negative” factors; somatic complaints, depressive affect and interpersonal problems, are closely correlated.

As expected, the scores of CES-D and all subscales were higher in those with depression symptoms by psychiatrists’ interview than those without depression symptoms, suggesting good discrimination in detecting depression symptoms from non-depression symptoms. This finding supports a validation study of CES-D among pregnant women with and without HIV where CES-D among those with depression symptoms was higher [16]. The performance characteristics for the Vietnamese translation of the CES-D in this sample from Vietnam (AUC = 0.88) were better than those reported for other language versions. For example, in a South African sample, the AUC was 0.76 [47]. For comparison with studies employing the CES-D both in general populations and HIV positive patients, the commonly used cut-off points, 16 (mild) and 21 (moderate), were used. Using the cut-off of 16, this study identified 36.5 % PLHIV as having symptoms of depression compared to 24.3 % in the Vietnamese general population [25], 16 % in the Australian general population [48], and 20 % in HIV positive patients in the US [39]. Using the cut-off of 21, this study identified 19.8 % PLHIV with symptoms of depression compared to 13 % in the Vietnamese general population [25] and 11 % among HIV positive patients in the US [39]. Although 16 was shown to be the optimal cutoff, users of the Vietnamese version of the CES-D should also take into account the change of sensitivity and specificity and the percentage of agreement with the psychiatrists’ interview in determining the cut-off to use for decision making. For example, for screening purposes in Vietnamese OPCs the cut-off of 16 could be used to identify those needing referral to specialist mental health services.

Limitations of this study must be acknowledged. Participants in this study were classified as having symptoms of depression by psychiatrists following interview and may not necessarily have met full criteria for Major Depressive Disorder. Instead, psychiatrists were asked to identify participants who had sufficient symptoms of depression to warrant referral to a specialist service. However, the aim of the study was to validate the scale for the purpose of referral to services where further assessment and diagnosis could be made. This study has not examined the test-retest reliability of the CES-D. As the CES-D measures symptoms over the past seven days, the person may not be experiencing depression but only low mood and many of these symptoms may occur in normal daily life. Further, the internal consistency might be affected by a range of participant characteristics that vary over time and thus establishing the test-retest reliability could be helpful. Finally, the sample was drawn from outpatient clinics in a very large Vietnamese city and might not be generalizable to PLHIV in other settings such as hospitals or other areas of Vietnam. PLHIV at hospitals are normally in a severe HIV/AIDS condition and might have higher prevalence of depression than outpatients. Further work is necessary to assess the psychometric properties of the scale in other areas and among PLHIV in other settings.

## Conclusions

The findings contribute to the international validation of the CES-D in screening for depression in HIV positive individuals. Together with previous studies, the scale was shown to be acceptable, reliable and valid for screening in Vietnamese HIV outpatient clinic settings where mental health specialists are not always available. Further research examining the psychometric properties utilizing different methods such as test-retest or in different settings such as HIV outpatient clinics in rural areas or of hospitalized PLHIV is needed.

### Ethics approval and consent to participate

All procedures performed in studies involving human participants were in accordance with the ethical standards of the Human Ethics Committee at Ho Chi Minh City Provincial AIDS Committee, Vietnam (Approval number: IRB-03-2013, dated 17/10/2013) and the University of Sydney, Australia (Approval number: 2013/859, dated 15/11/2013). Written informed consent was obtained from all individual participants included in the study.

### Consent for publication

Not applicable.

### Availability of data and materials

Available upon request to the first author Thai Thanh Truc: thaithanhtruc@fphhcm.edu.vn.

## Additional file

Additional file 1: Vietnamese version of the Center for Epidemiologic Studies – Depression scale. (DOCX 17 kb)

## Abbreviations

CES-D: center for epidemiologic studies – depression scale
CFI: comparative fit indices
GFI: goodness of fit indices
IFI: incremental fit indices
OPC: outpatient clinic
PLHIV: people living with HIV/AIDS
RMSEA: root mean square error of approximation
