# Supplementary material for: Screening value of the Center for epidemiologic studies – depression scale among people living with HIV/AIDS in Ho Chi Minh City, Vietnam: a validation study
Source: BMC Psychiatry. 2016 May 13;16:145. doi: 10.1186/s12888-016-0860-3 (PMC4868017; doi:10.1186/s12888-016-0860-3)
Supplement: Additional file 1: — Vietnamese version of the Center for Epidemiologic Studies – Depression scale. (DOCX 17 kb) [file 12888_2016_860_MOESM1_ESM.docx]

Additional file 1: Vietnamese version of the Center for Epidemiologic Studies – Depression scale [1]

**Các câu dưới đây là về cảm nhận của bạn trong tuần vừa qua (7 ngày qua). Vui lòng khoanh tròn con số thể hiện cảm nhận của bạn.**

| **Trong suốt tuần vừa qua (7 ngày),** | | **Hiếm khi hoặc không khi nào**  **(<1 ngày)** | **Đôi khi hoặc một vài lần**  **(1-2 ngày)** | **Thỉnh thoảng hoặc nhiều lần (3-4 ngày)** | **Hầu hết hoặc tất cả thời gian**  **(5-7 ngày)** |
| --- | --- | --- | --- | --- | --- |
| 1 | Trong tuần qua, tôi buồn bực vì những điều thường ngày không gây bực cho tôi | 0 | 1 | 2 | 3 |
| 2 | Trong tuần qua, tôi cảm thấy không muốn ăn và ăn không thấy ngon | 0 | 1 | 2 | 3 |
| 3 | Trong tuần qua, tôi cảm thấy tôi không thể xua tan nỗi buồn dù gia đình, bạn bè giúp đỡ tôi | 0 | 1 | 2 | 3 |
| 4 | Trong tuần qua, tôi cảm thấy tôi cũng tốt lành/khỏe mạnh như những người khác | 0 | 1 | 2 | 3 |
| 5 | Trong tuần qua, tôi có vấn đề trong việc ghi nhớ việc đang làm | 0 | 1 | 2 | 3 |
| 6 | Trong tuần qua, tôi cảm thấy bị suy nhược/trầm cảm | 0 | 1 | 2 | 3 |
| 7 | Trong tuần qua, tôi thấy mọi việc tôi làm đều là sự gắng sức | 0 | 1 | 2 | 3 |
| 8 | Trong tuần qua, tôi cảm thấy tràn đầy hi vọng về tương lai | 0 | 1 | 2 | 3 |
| 9 | Trong tuần qua, tôi cảm thấy cuộc đời tôi từ trước đến nay toàn là thất bại | 0 | 1 | 2 | 3 |
| 10 | Trong tuần qua, tôi thấy sợ hãi | 0 | 1 | 2 | 3 |
| 11 | Trong tuần qua, tôi ngủ không yên giấc | 0 | 1 | 2 | 3 |
| 12 | Trong tuần qua, tôi vui vẻ | 0 | 1 | 2 | 3 |
| 13 | Trong tuần qua, tôi nói chuyện ít hơn bình thường | 0 | 1 | 2 | 3 |
| 14 | Trong tuần qua, tôi cảm thấy cô đơn | 0 | 1 | 2 | 3 |
| 15 | Trong tuần qua, mọi người đã không thân thiện | 0 | 1 | 2 | 3 |
| 16 | Trong tuần qua, tôi yêu thích cuộc sống | 0 | 1 | 2 | 3 |
| 17 | Trong tuần qua, tôi đã khóc nức nở | 0 | 1 | 2 | 3 |
| 18 | Trong tuần qua, tôi cảm thấy buồn | 0 | 1 | 2 | 3 |
| 19 | Trong tuần qua, tôi cảm thấy mọi người ghét tôi | 0 | 1 | 2 | 3 |
| 20 | Trong tuần qua, tôi không thể bắt đầu làm việc gì | 0 | 1 | 2 | 3 |

[1] Radloff LS. The CES-D Scale: **A Self-Report Depression Scale for Research in the General Population**. *Applied Psychological Measurement* 1977, **1**(3):385-401.
